# Effect of GOLPH3 on cumulus granulosa cell apoptosis and ICSI pregnancy outcomes

**DOI:** 10.1038/s41598-017-08343-w

**Published:** 2017-08-11

**Authors:** Dianliang Lin, Jing Ran, Suqin Zhu, Song Quan, Baofeng Ye, Aili Yu, Yuefan Kang, Yuan Lin

**Affiliations:** 1 Fujian Provincial Reproductive Medicine Center, Fujian Provincial Maternity & Children Hospital, Affiliated Hospital of Fujian Medical University, No. 18 Daoshan Road, Fuzhou City, Fujian Province 350001 China; 2 Center for Reproductive Medicine, Department of Obstetrics and Gynecology, Nanfang Hospital, Southern Medical University, 1838 North Guangzhou Road, Guangzhou City, Guangdong Province 510515 China; 3 Department of Gynecology and Obstetrics, the First Affiliated Hospital of Xiamen University, 55 Zhenhai Road, Xiamen City, Fujian Province 361003 China

## Abstract

Previous studies have shown that GOLPH3 mediates cell growth, proliferation and differentiation and inhibits cell apoptosis; however, the role of GOLPH3 in cumulus granulosa cells and the value of GOLPH3 in predicting ICSI pregnancy outcomes remain unknown until now. Our findings showed higher positive expression rate, score of staining intensity, and immunohistochemical score of GOLPH3 in the cumulus granulosa cells of the pregnant women relative to non-pregnant women, and a higher apoptotic rate of cumulus granulosa cells was detected in non-pregnant women than in pregnant women. Pearson correlation analyses revealed that pregnancy correlated negatively with GOLPH3 expression and apoptosis of cumulus granulosa cells, and positively with the number of follicles punctured, number of grade III oocytes, number of eggs retrieved for ICSI, number of zygotes, number of cleavage-stage embryos, number of top-quality embryos, number of blastocysts, number of top-quality blastocysts, and number of frozen embryos. GOLPH3 may be involved in the apoptosis of cumulus granulosa cells, which may correlate with oocyte maturation and egg development. GOLPH3 expression in cumulus granulosa cells may facilitate the selection of top-quality eggs and embryos, the prediction of the clinical pregnancy outcomes of ICSI, and the increase of the pregnancy rate.

## Introduction

Intracytoplasmic sperm injection (ICSI) procedure involving injection of a single sperm directly into a human egg under a microscope, is predominantly used for the treatment of male factor infertility^1^. The continuous improvements in controlled ovarian hyperstimulation (COHS), follicular monitoring, identification of top-quality embryos and embryo transfer procedures result in a remarkable rise in the pregnancy rate following cleavage embryo or blastocyst transfer in subjects undergoing ICSI; however, there are still 40 to 50% patients that fail in pregnancy^2^. Since the improvement of the egg quality may increase the implantation rate and pregnancy rate of ICSI-fertilized embryos, an accurate evaluation of the egg quality and selection of eggs with top quality and developmental potential for ICSI, is therefore of great importance in assisted reproductive technology (ART)^3^.

Cumulus granulosa cells, a group of closely associated granulosa cells that surround and nourish oocytes, are an important mediator to regulate oocyte development^4^. In addition, cumulus granulosa cells may preserve and release some growth factors and specific proteins, which are sequentially expressed or selectively diffused during oocyte maturation and post-fertilization embryo development to mediate egg and embryo development^5^.

Pro-apoptotic and anti-apoptotic factors have been found to play important roles in follicular growth, selection and atresia^6^, and granulosa cells are reported to affect oocyte quality^7^. It has been demonstrated that the loss of germ cells initiates from the apoptosis of granulosa cells; however, oocyte apoptosis is a beginning of follicular atresia, while apoptosis of follicular granulosa cells is the root cause of follicular degeneration^8^. During follicular development, granulosa cell apoptosis may cause follicular atresia^9^. Therefore, apoptosis of granulosa cells is considered as an indicator for the developmental potential of oocytes^10^. It is reported that egg maturation, fertilization and the quality of the resultant embryos are strongly associated with the apoptosis of cumulus granulosa cells^11, 12^, while cumulus granulosa cell apoptosis has been found to correlate with egg fertilizing ability, and patients’ age, number of eggs obtained, fertilization rate and pregnancy outcomes after *in vitro* fertilization (IVF)^13^. It is therefore considered that the apoptosis of cumulus granulosa cells may facilitate the ability of egg development and predict the pregnancy outcomes after IVF or ICSI. However, a high apoptotic rate of granulosa cells, notably cumulus granulosa cells surrounding oocytes, may cause follicular development disorder and directly affect egg quality, thereby resulting in a decline in the ability of oocyte development^14^. It is therefore of urgent need to investigate the key molecules mediating granulosa cell apoptosis and the underlying mechanisms, develop approaches to reduce ovarian granulosa cell apoptosis and suppress granulosa cell apoptosis and enhance egg developmental potential, to screen top-quality eggs for ICSI/IVF and increase embryo quality through the treatment of egg and embryo development at a molecular level, thereby resulting in an increase in the success rate of IVF.

Golgi phosphoprotein 3 (GOLPH3), also known as GOPP1, GPP34, MIDAS and Vps74, is localized on human chromosome 5p13, which is found to mediate cell growth, proliferation and differentiation and inhibit cell apoptosis^15^. In cancer cells, elevation of GOLPH3 expression causes a clear-cut enlargement of cell volume and acceleration of cell division, while inhibition of GOLPH3 expression results in a reduction of cell size^16^. In addition, GOLPH3 was found to be involved in the growth, differentiation and proliferation of cancer cells via mammalian target of rapamycin (mTOR) signaling^17^. This protein may activate rapamycin-sensitive and -insensitive complexes, which induces an increase in intracellular p70S6K and serine/threoninekinase (Akt) activities; while activated Akt acts on Caspase-9 to allow its phosphorylation to cause Caspase-9 inactivation, thereby suppressing pro-apoptosis^18^. As an apoptosis initiator, Caspase-9 inactivation may block the activation of the apoptosis executor Caspase-3, which finally inhibits apoptosis, accelerates protein synthesis, increases the production of intracellular factors mediating protein synthesis and promotes cell division positively^19^.

To our knowledge, however, there is no report on the role of GOLPH3 in follicular growth, selection and atresia, GOLPH3 expression in cumulus granulosa cells, the effect of GOLPH3 on granulosa cell apoptosis and oocyte development or the value of GOLPH3 in predicting the pregnancy outcomes of ICSI to date. The present study was therefore designed with aims to evaluate the effect of GOLPH3 on the ability of oocyte development and ICSI pregnancy outcomes and investigate the mechanisms underlying the regulation of top-quality eggs and top-quality embryo formation, so as to provide theoretical and experimental evidence for increasing the success rate of pregnancy through the interventions on egg and embryo quality.

## Results

### Comparison of demographic and clinical characteristics between pregnant and non-pregnant women after ICSI

Among the 119 subjects undergoing ICSI, 65 women with successful pregnancy were assigned to the pregnant group, and 54 women with failure in pregnancy were assigned to the non-pregnant group according to the follow-up outcomes. The demographic and clinical characteristics of pregnant and non-pregnant women are described in Table 1. There were no significant differences seen in female age, male age, BMI, duration of sterility, FSH, LH, E_2_, PRL, T, FSH/LH, number of baseline antral follicles in left or right ovary, mean Gn concentration, mean duration of Gn per cycle, or mean endometrial thickness between the pregnant and non-pregnant groups (all *P* values > 0.05).

**Table 1:** Comparison of demographic and clinical characteristics between pregnant and non-pregnant women after ICSI.

	Parameter	Non-pregnant group (*n* = 54)	Pregnant group (*n* = 65)	*P* value
	Female age (years)	29.20 ± 2.43	28.98 ± 3.20	0.680
	Male age (years)	31.78 ± 3.53	32.23 ± 3.95	0.515
	BMI (kg/m^2^)	20.49 ± 2.95	20.16 ± 2.79	0.532
	Duration of sterility (years)	3.93 ± 1.83	4.45 ± 2.08	0.154
Type of sterility	Primary sterility	49	48	
	Secondary sterility	15	17	
	Cause of sterility			
Female factor	Tubal factor	12	10	
Male factor	Oligospermia	2	0	
	Asthenospermia	5	7	
	Teratozoospermia	10	15	
	Oligoasthenozoospermia	11	12	
	Oligoteratozoospermia	3	4	
	Asthenoteratozoospermia	17	20	
	Oligoasthenoteratozoospermia	6	7	
Baseline hormone level	FSH (mIU/ml)	5.85 ± 1.32	5.91 ± 1.57	0.813
	LH (mIU/ml)	3.69 ± 1.47	3.94 ± 1.74	0.414
	E_2_ (pmol/L)	41.04 ± 17.41	44.95 ± 24.11	0.321
	PRL (ng/ml)	16.34 ± 12.53	17.22 ± 10.95	0.516
	T (nmol/ml)	0.39 ± 0.14	0.36 ± 0.16	0.686
	FSH/LH	1.79 ± 0.68	1.88 ± 1.19	0.622
No. of baseline antral follicles	Left ovary	9.18 ± 3.55	9.05 ± 3.19	0.823
	Right ovary	10.48 ± 3.92	10.29 ± 3.92	0.786
Mean Gn level (IU)		2124.54 ± 423.36	2073.46 ± 507.59	0.557
Duration of Gn per cycle (day)		6.48 ± 1.87	6.42 ± 3.25	0.895
Mean endometrial thickness (mm)		11.64 ± 1.45	11.62 ± 1.98	0.941

### Comparison of clinical outcomes between pregnant and non-pregnant women after ICSI

A total of 119 ICSI cycles were performed, and each subject was transferred with 2 embryos, with an implantation rate of 30.25%. Among the 65 pregnant women (54.62% pregnancy rate), there were 61 cases with clinical pregnancy, including 5 early abortions (8.2%); 2 cases with ectopic pregnancy (3.08%); and 2 cases with biochemical pregnancy. The clinical outcomes of pregnant and non-pregnant women following ICSI are shown in Table 2. There were no significant differences in the number of grade II oocytes, number of eggs retrieved per cycle, rate of egg retrieval, fertilization rate, or rate of cleavage-stage embryo between the pregnant and non-pregnant women (all *P* values > 0.05), while more follicles punctured, grade III oocytes, eggs retrieved for ICSI, zygotes, cleavage-stage embryos, top-quality embryos, blastocysts, top-quality blastocysts, frozen embryos and higher blastocyst formation rate and rate of frozen embryos were detected in the pregnant group than in the non-pregnant group (all *P* values < 0.05), suggesting higher egg and embryo quality in pregnant women than in non-pregnant women.

**Table 2:** Comparison of clinical outcomes between pregnant and non-pregnant women after ICSI.

Parameter	Non-pregnant group (*n* = 54)	Pregnant group (*n* = 65)	*P* value
No. of follicles punctured	13.44 ± 4.55	15.32 ± 4.95	0.035
No. of grade II oocytes	5.72 ± 3.18	5.97 ± 3.40	0.685
No. of grade III oocytes	5.87 ± 3.07	7.23 ± 3.16	0.020
No. of eggs retrieved per cycle	11.59 ± 4.23	13.20 ± 4.72	0.055
Rate of egg retrieval (%)	86.23(626/726)	86.14(858/996)	0.240
No. of eggs retrieved for ICSI	10.02 ± 3.54	11.72 ± 4.11	0.018
No. of zygote	8.48 ± 2.99	10.25 ± 3.56	0.005
Fertilization rate (%)	84.50(458/542)	88.10(666/756)	0.061
No. of cleavage-stage embryo	7.94 ± 2.99	9.63 ± 3.44	0.006
Rate of cleavage-stage embryo (%)	93.66(429/458)	93.99(626/666)	0.823
Mean No. of top-quality embryo	4.80 ± 2.85	6.28 ± 2.85	0.006
Mean No. of blastocyst	1.96 ± 1.69	3.21 ± 1.81	0.000
No. of top-quality blastocyst	1.28 ± 1.51	2.03 ± 1.54	0.008
Blastocyst formation rate (%)	45.11(106/235)	57.89(209/361)	0.002
Mean No. of frozen embryos	1.59 ± 1.65	1.59 ± 1.65	0.002
Rate of frozen embryos	62.96 (34/54)	92.30(60/65)	0.000

### Comparison of GOLPH3 expression in cumulus granulosa cells between pregnant and non-pregnant women after ICSI

**Figure 1:**
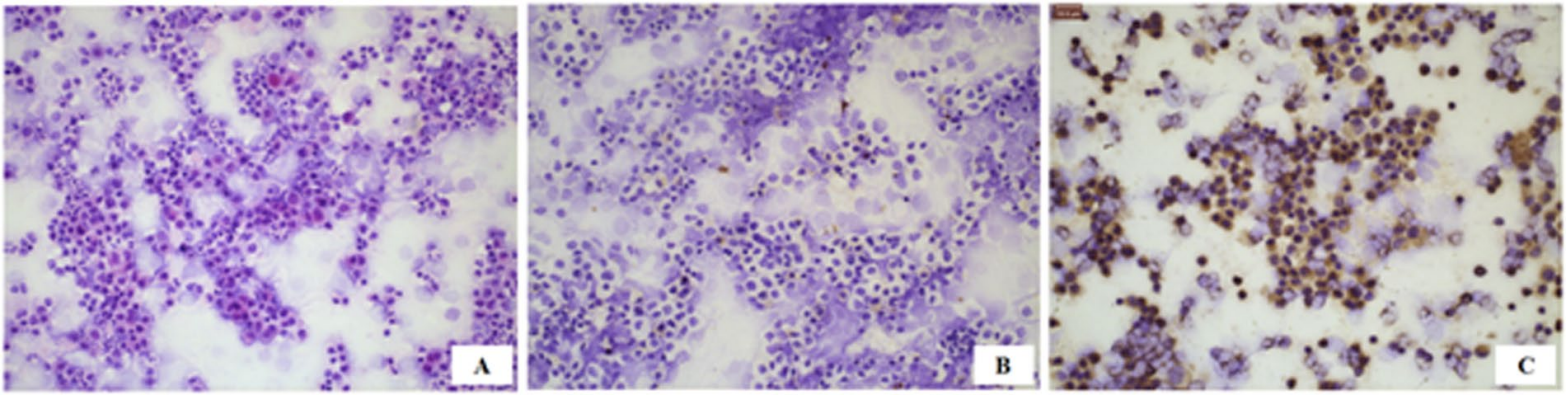
Immunohistochemical staining of GOLPH3 in cumulus granulosa cells. (**A**) HE staining of cumulus granulosa cells, ×20; (**B**) Negative GOLPH3 expression in cumulus granulosa cells with DAB staining and hematoxylin counterstaining, and PBS serves as the primary antibody, ×20; (**C**) Positive GOLPH3 expression in cumulus granulosa cells with DAB staining and hematoxylin counterstaining, ×20.

Immunohistochemical staining was performed to determine GOLPH3 protein expression in 119 specimens of cumulus granulosa cells. GOLPH3 was found to be predominantly expressed in cytoplasm (Fig. 1), with a positive expression rate of 3 to 57% (mean, 29.13% ± 14.97%) and an immunohistochemical score from 1 to 9 (mean, 4.57 ± 2.38). Significantly higher positive expression rate (40.46% ± 4.53% vs. 15.50% ± 6.51%), score of positive expression rate (2.25 ± 0.43 vs. 1.81 ± 0.39), score of staining intensity (2.67 ± 0.59 vs. 1.50 ± 0.54), and immunohistochemical score (6.09 ± 2.01 vs. 2.74 ± 1.20) of GOLPH3 were detected in the cumulus granulosa cells of pregnant women than in non-pregnant women (all *P* value < 0.01), indicating that the ICSI pregnancy outcome correlates with GOLPH3 expression in cumulus granulosa cells.

### Comparison of cumulus granulosa cell and sperm apoptosis between pregnant and non-pregnant women after ICSI

**Figure 2:**
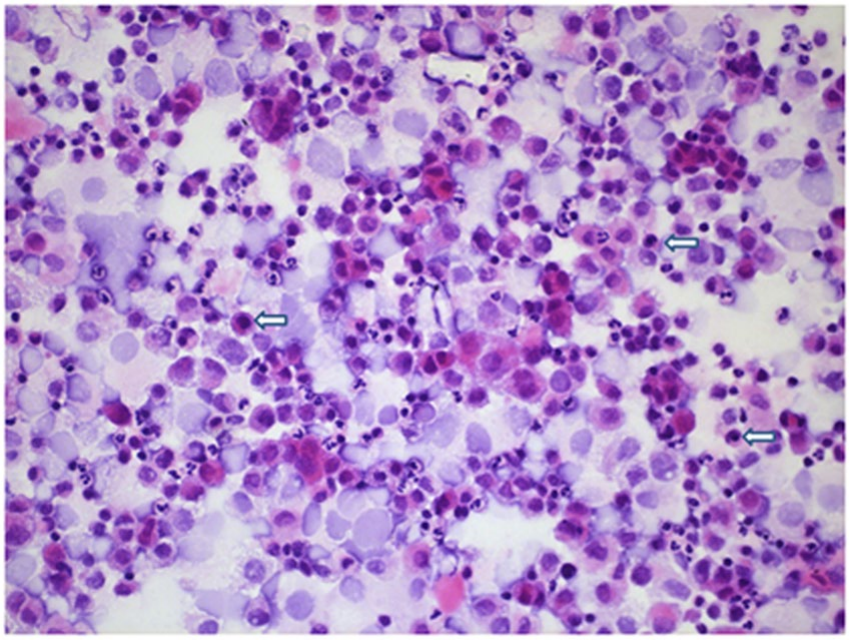
Apoptosis of cumulus granulosa cells (HE staining, ×100). The arrows indicate pyknosis of cumulus granulosa cells.

**Figure 3:**
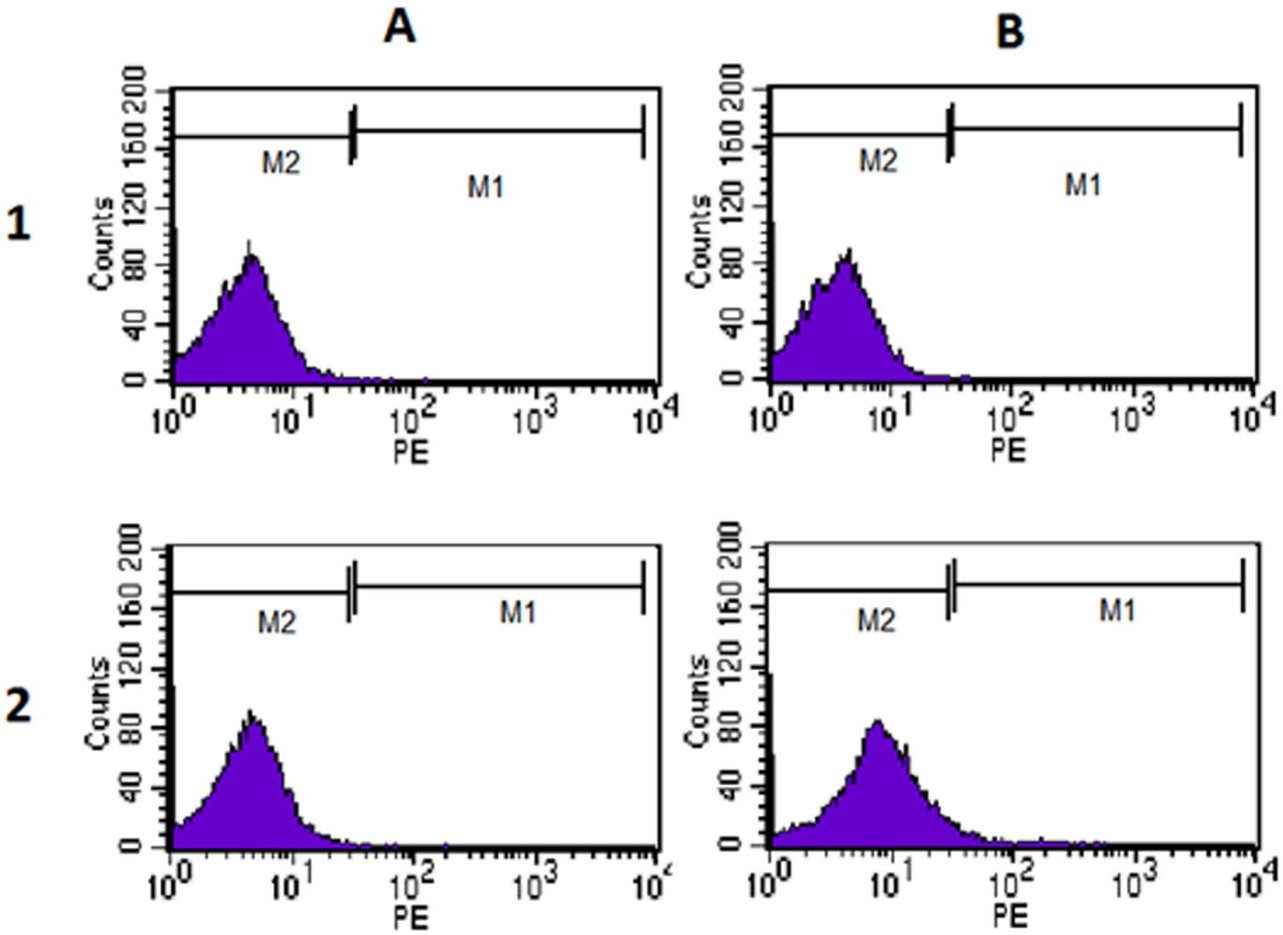
Activated Caspase-3 expression in cumulus granulosa cells by flow cytometry. (**A**) PBS serves as the primary antibody; (**B**) Positive expression of activated Caspase-3. 1, Non-pregnant group; 2, Pregnant group;

HE staining showed karyopyknosis of cumulus granulosa cells, and shrinking and accumulation of cell nucleus, with deep blue staining (Fig. 2), indicating DNA transcription termination and cumulus granulosa cell apoptosis. Of the 119 specimens of cumulus granulosa cells detected, the mean apoptotic rate was 16.11% ± 12.04% (range, 0.21 to 35.23%), and a higher apoptotic rate of cumulus granulosa cells was detected in non-pregnant women than in pregnant women (27.52% ± 5.32% vs. 6.62% ± 6.5%, *P* < 0.01). Flow cytometry detected 0.77% ± 0.17% (range, 0.01 to 13.06%) expression of activated Caspase-3 in cumulus granulosa cells (Fig. 3), and a significantly greater expression rate of activated Caspase-3 was detected in non-pregnant women than in pregnant women (1.61% ± 2.34% vs. 0.07% ± 0.07%, *P* < 0.01), suggesting that the ICSI pregnancy outcome is associated with the apoptosis of cumulus granulosa cells.

In addition, 0.01 to 1.57% (mean, 0.36% ± 0.37%) expression rates of activated Caspase-3 were detected in sperm, and no significant difference was seen in the expression rate of activated Caspase-3 in the sperm between the pregnant and non-pregnant groups (0.33% ± 0.36% vs. 0.39% ± 0.39%, *P* > 0.05).

### Association of pregnancy with GOLPH3 expression, cumulus granulosa cell apoptosis and ICSI clinical outcomes

Pearson correlation analyses revealed that pregnancy correlated negatively with GOLPH3 expression (*r* = −0.946, *P* < 0.01) and cumulus granulosa cell apoptosis (*r* = −0.557, *P* < 0.01), and positively with the number of follicles punctured (*r* = 0.34, *P* < 0.01), number of grade III oocytes (*r* = 0.27, *P* < 0.01), number of eggs retrieved for ICSI (*r* = 0.412, *P* < 0.01), number of zygotes (*r* = 0.486, *P* < 0.01), number of cleavage-stage embryos (*r* = 0.492, *P* < 0.01), number of top-quality embryos (*r* = 0.557, *P* < 0.01), number of blastocysts (*r* = 0.699, *P* < 0.01), number of top-quality blastocysts (*r* = 0.53, *P* < 0.01), and number of frozen embryos (*r* = 0.593, *P* < 0.01).

### Comparison of GOLPH3 expression in cumulus granulosa cells between pregnant and non-pregnant women after ICSI

**Figure 4:**
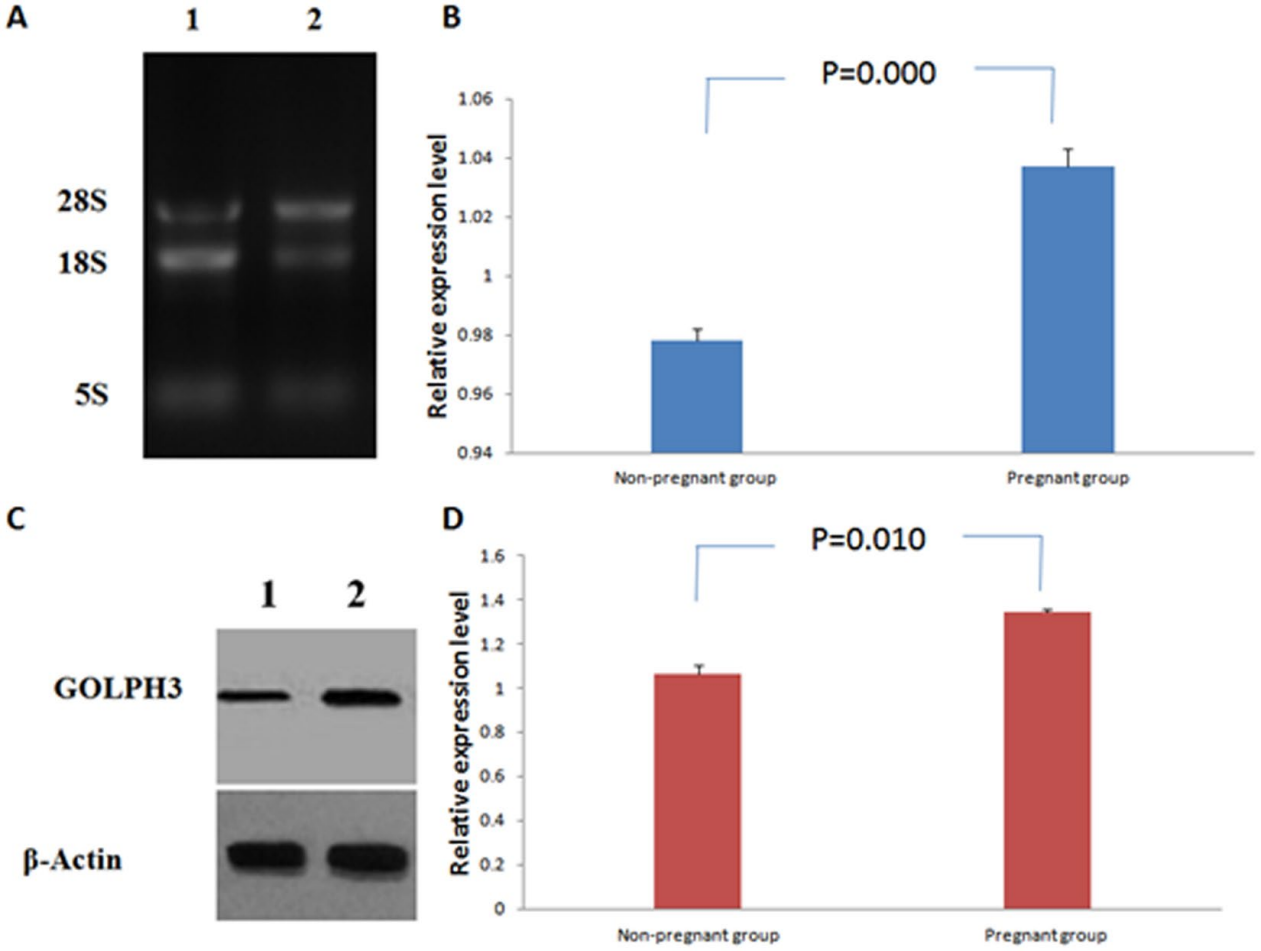
qRT-PCR and Western blotting assays detect GOLPH3 expression in cumulus granulosa cells. (**A**) Agarose gel electrophoresis of total *GOLPH3* RNA. 1, Non-pregnant group; 2, Pregnant group; (**B**) Comparison of relative *GOLPH3 mRNA* expression in cumulus granulosa cells between the pregnant- and non-pregnant groups; (**C**) Western blotting analysis detects GOLPH3 protein expression in cumulus granulosa cells, and β-actin serves as a loading control. 1, Non-pregnant group; 2, Pregnant group; (**D**) Comparison of GOLPH3 protein expression in cumulus granulosa cells between the pregnant- and non-pregnant groups.

qRT-PCR assay detected higher *GOLPH3 mRNA* expression in cumulus granulosa cells in pregnant women than in non-pregnant women after ICSI (1.04 ± 0.01 vs. 0.98 ± 0.003, *P* < 0.01), and higher GOLPH3 protein expression was observed in cumulus granulosa cells in pregnant women than in non-pregnant women after ICSI (1.34 ± 0.01 vs. 1.06 ± 0.11, *P* < 0.05), as revealed by Western blotting analysis (Fig. 4; Supplementary Fig. 1).

### ICSI fertilization and embryo development after retrieval of cumulus granulosa cells from single oocytes

**Figure 5:**
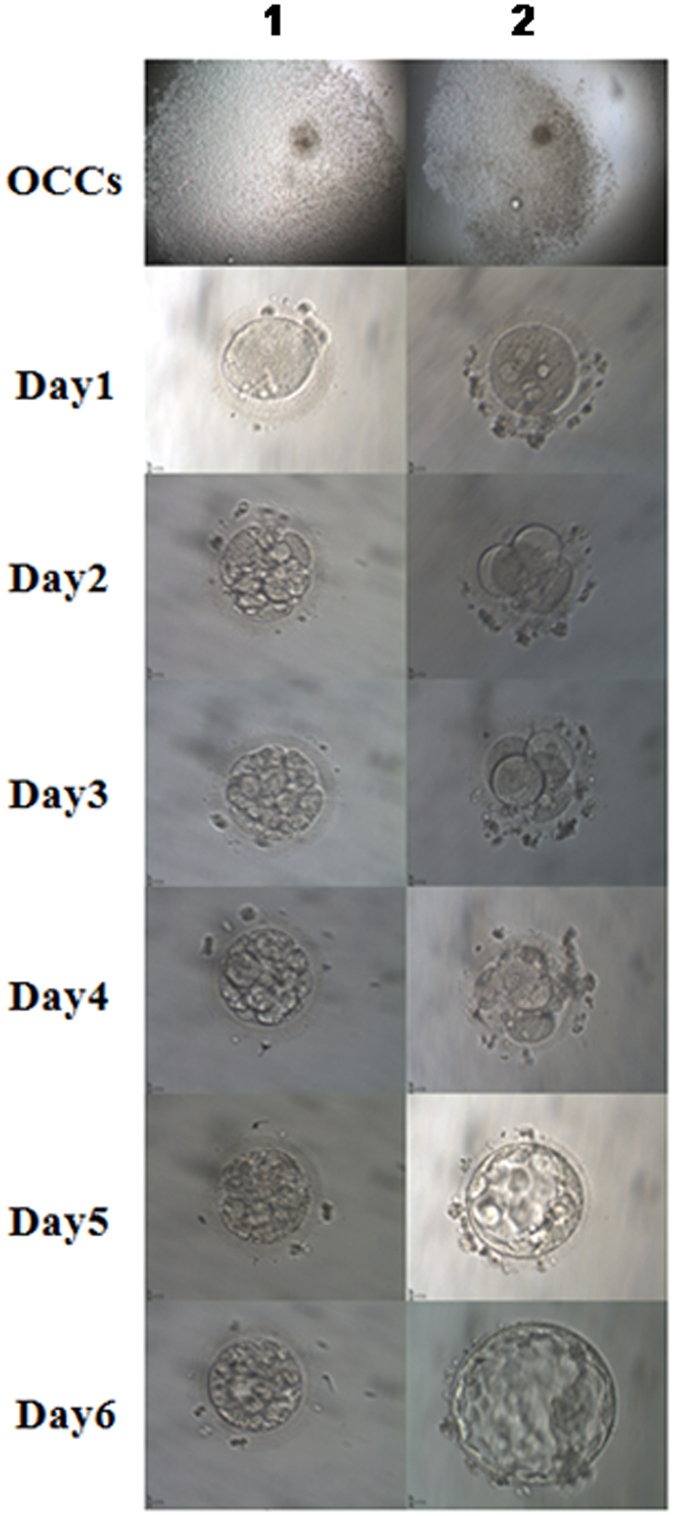
ICSI fertilization and embryo development.OCCCs, ×4; Oocytes, Day 1 to 6 embryos, ×20; 1, Non-blastocyst group; 2, Blastocyst group.

After cumulus granulosa cells were collected from 347 eggs, the embryo development and blastocyst formation were observed in each fertilized egg after ICSI (Fig. 5). Of the 347 eggs observed, 98 eggs developed blastocysts after ICSI, with a blastocyst formation rate of 28.24%, including 69 day 5 blastocysts (70.41%) and 29 day 6 blastocysts (29.51%).

### Comparison of GOLPH3 expression and apoptosis of cumulus granulosa cells between non-blastocyst and blastocyst groups

**Figure 6:**
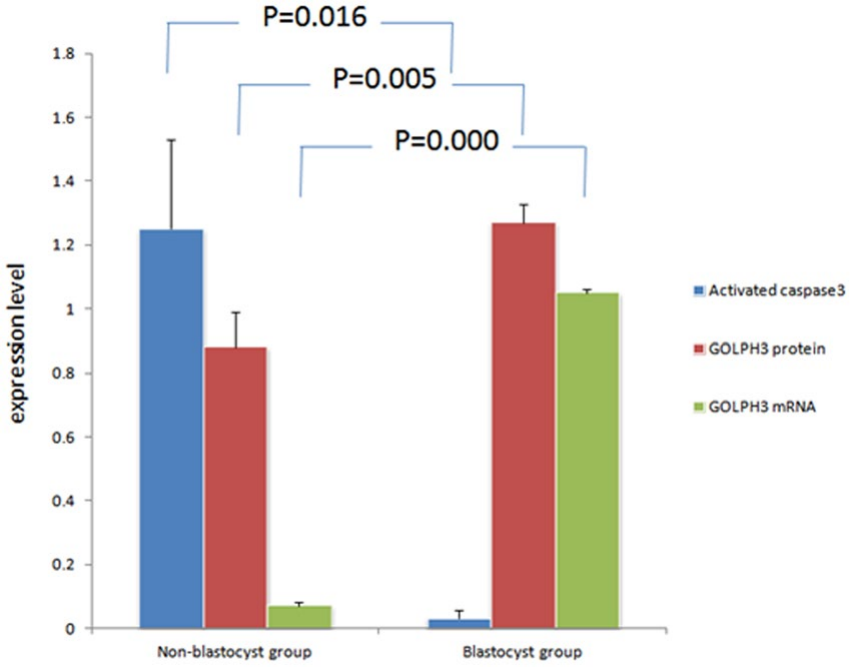
Comparison of cumulus granulosa cell apoptosis and GOLPH3 expression in cumulus granulosa cells between the non-blastocyst and blastocyst groups.

The cumulus granulosa cells were harvested from the peripheral regions of the 98 eggs developing blastocysts and 249 eggs without development of blastocysts. The apoptosis of cumulus granulosa cells was detected by measuring the expression of activated Caspase-3 using flow cytometry, and the GOLPH3 expression was determined in cumulus granulosa cells with Western blotting and qRT-PCR assays at translational and transcriptional levels. Flow cytometry revealed a higher apoptotic rate of cumulus granulosa cells in the non-blastocyst group than in the blastocyst group (1.25 ± 0.28 vs. 0.03 ± 0.03, *P* < 0.01), and higher GOLPH3 expression was detected in cumulus granulosa cells in the blastocyst group relative to the non-blastocyst group at both translational and transcriptional levels (protein expression, 1.27 ± 0.06 vs. 0.88 ± 0.11, *P* < 0.01; *mRNA* expression, 1.05 ± 0.01 vs. 0.07 ± 0.01, *P* < 0.01) (Fig. 6).

## Discussion

Since ICSI was introduced in 1992^20^, this technique has become an effective approach for the treatment of severe male infertility^1^. Although transfer of cleavage-stage embryos or blastocysts results in a clear-cut success rate of ICSI pregnancy, there are still approximately a half of the individuals undergoing ICSI that fail in pregnancy^2, 3^. In the current study, a total of 119 ICSI cycles were performed, and we observed a 86.18% egg retrieval rate, 86.27% fertilization rate, 93.86% percentage of cleavage-stage embryos, 63.22% proportion of top-quality embryos, 52.85% blastocyst formation rate, 63.81% percentage of top-quality blastocysts, 78.99% percentage of frozen embryos. There were 2 embryos transferred to each subject, which resulted in a 30.25% implantation rate and 54.62% pregnancy rate, indicating that there are still 45.38% women with failure in pregnancy after ICSI.

The selection of top-quality eggs with high developmental potential is of great importance in ICSI, since the improvement of egg quality may increase the implantation rate and pregnancy rate of ICSI fertilized eggs^21, 22^. The development ability of oocytes, also known as oocyte quality, determines the success rate of retrieving *in vitro* cultured top-quality embryos^23, 24^. Although great advances have been achieved in *in-vitro* culture techniques^25^, the efficiency of producing top-quality embryos remains low, and only 30 to 40% cleavage-stage embryos develop to blastocysts^26^, which is far less than the 85% blastocyst formation rate under the *in-vivo* conditions^27^. Such a low efficiency is reported to be associated with the culture medium^28^, and it has been demonstrated that the alteration of the medium may affect the development ability of early-stage embryos and increase the blastocyst formation rate in a limited manner^29^. However, oocyte quality is the primary factor determining the blastocyst formation rate^30, 31^. Therefore, effective retrieval of top-quality oocytes with developmental ability is critical to producing top-quality embryos and facilitating blastocyst formation^32^. In the present study, the pregnant women were found to have significantly more follicles punctured, more grade III oocytes and more eggs retrieved for ICSI than non-pregnant women, highlighting the superiority of egg maturation and quality, and more zygotes, cleavage-stage embryos, top-quality embryos, blastocysts, top-quality blastocysts, frozen embryos and higher blastocyst formation rate and rate of frozen embryos were detected in the pregnant group than in the non-pregnant group (all *P* values < 0.05), which was in agreement with previous reports. It is suggested that the development ability or quality of oocytes may be evaluated using the egg-related laboratory parameters after ICSI.

In an antral follicle, cumulus granulosa cells surround oocyts and mediate oocyte maturation and development through complicated junctional mechanisms^4^. In addition, cumulus granulosa cells preserve and release growth factors and specific proteins, which are found to regulate egg and embryo development^4^. However, cumulus granulosa cells are usually removed and discarded during ICSI, and these discarded cells may contain important information pertaining to oocyte development and maturation^33^. It is therefore considered that detection of gene expression in cumulus granulosa cells may reflect the alteration of follicle microenvironment and track oocyte development-associated information, which facilitates the indirect assessment of egg quality and embryo development potential. Moreover, analyses of cumulus granulosa cells and a search for molecular markers with predictive values of egg quality and embryo development potential may provide new insights into the selection of embryos^33^. In the current study, 119 specimens of cumulus granulosa cells after ICSI and 347 specimens of cumulus granulosa cells surrounding the single egg were retrieved to investigate GOLPH3 expression in cumulus granulosa cells and examine the effect of cumulus granulosa cell apoptosis on the development ability of eggs. Our findings demonstrate that cumulus granulosa cells contain a great deal of information associated with the development ability of eggs and pregnancy outcomes.

Granulosa cell apoptosis has been identified as the primary factor leading to follicular atresia, and has been found to affect oocyte structure and oocyte developmental potential^34–36^. Oocytes with a low apoptotic rate of cumulus granulosa cells have normal morphology^37^, while those with a high apoptotic rate of cumulus granulosa cells have abnormal morphology, including mitochondrial relocation^38^, presence of secondary lysosome^39^, abnormal morphology of the first polar body^4^, and abnormal zona pellucida morphology^40^. Previous studies have shown that a rise in the apoptotic rate of cumulus granulosa cells, a reduction in intracellular junction, and a decrease and even disappearance of microvilli may cause low maturation and abnormal morphology of oocytes, which affects oocyte development, thereby affecting IVF or ICSI pregnancy outcomes^41^. It was reported that egg maturation, fertilization, and the quality of the corresponding embryos were closely associated with the apoptosis of cumulus granulosa cells^11^, and egg-fertilizing ability was also found to correlate with cumulus granulosa cell apoptosis^12^. In addition, the apoptotic rate of cumulus granulosa cells has been demonstrated to correlate with patients’ age, number of oocytes retrieved, fertilization rate and IVF pregnancy outcomes^13^. In the current study, we observed 0.21 to 35.23% (mean, 16.11% ± 12.04%) apoptotic rates of cumulus granulosa cells, and flow cytometry detected 0.01 to 13.06% (mean, 0.77% ± 0.17%) expression rate of activated Caspase-3 in cumulus granulosa cells. As a key executor, Caspase-3 functions in multiple apoptotic signaling pathways. Caspase-3 exists as an inactive 32 kD proenzyme, and is activated at early-stage apoptosis; activated form of Caspase-3 is composed of a 17 kD subunit and a 12 kD subunit, leading to cleavage of cytoplasmic and nuclear substrates, which finally results in apoptosis^19^. In this study, we detected a significant difference in the apoptotic rate of cumulus granulosa cells between the pregnant- and non-pregnant women after ICSI, indicating the correlation between cumulus granulosa cell apoptosis and ICSI pregnancy outcomes. Moreover, a higher apoptotic rate of cumulus granulosa cells was observed in the non-blastocyst group than in the blastocyst group, demonstrating that the apoptosis of cumulus granulosa cells affect the development ability of oocytes and embryo quality. Elucidation of the mechanisms responsible for the apoptosis of cumulus granulosa cells may facilitate the development of approaches for inhibiting cumulus granulosa cell apoptosis and increasing egg developmental potential.

GOLPH3 has been shown to suppress apoptosis and be involved in cell growth, differentiation and proliferation, and pro-apoptosis and anti-apoptosis also occur during follicular growth, selection and atresia^15^. However, the role of GOLPH3 in cumulus granulosa cells remains unknown until now. In the current study, immunohistochemical staining revealed that GOLPH3 was predominantly localized in the cytoplasm of cumulus granulosa cells, and 3 to 57% (mean, 29.13% ± 14.97%) positive expression was detected in 119 cell specimens, with 4.57 ± 2.38 (range, 1 to 9) mean immunohistochemical staining scores. There were significant differences in the positive expression rate, staining intensity, and immunohistochemical staining score of GOLPH3 between the pregnant group and the non-pregnant group. Pearson correlation analysis revealed a remarkable decline in the apoptotic rate of cumulus granulosa cells with the elevation of GOLPH3 expression, and a clear-cut increase in the number of follicles punctured, number of grade III oocytes, number of eggs retrieved for ICSI, number of zygotes, number of cleavage-stage embryos, number of top-quality embryos, number of blastocysts, number of top-quality blastocysts, and number of frozen embryos with the rise in the GOLPH3 expression, suggesting the women with higher GOLPH3 expression in cumulus granulosa cells may have more top-quality oocytes for ICSI and elevated oocyte maturation and development ability. qRT-PCR and Western blotting assays showed higher GOLPH3 expression in cumulus granulosa cells at both transcriptional and translational levels in pregnant women than in non-pregnant women after ICSI, suggesting that elevated GOLPH3 expression may reduce apoptosis of cumulus granulosa cells and increase the development ability of oocytes, which finally affects the clinical pregnancy outcomes of ICSI.

Blastocyst formation is an indicator of a top-quality egg^42^. To evaluate the effect of GOLPH3 expression on blastocyst formation, we retrieved cumulus granulosa cells from the peripheral regions of the 98 eggs developing blastocysts and 249 eggs without development of blastocysts, and detected the apoptosis of and GOLPH3 expression in cumulus granulosa cells. Flow cytometry revealed a higher apoptotic rate of cumulus granulosa cells in the non-blastocyst group than in the blastocyst group, and higher GOLPH3 expression was detected in the blastocyst group than in the non-blastocyst group at both protein and mRNA levels.

Previous studies have shown that cumulus granulosa cells closely surround eggs and mediate oocyte maturation and development^43, 44^, and granulosa cell gene expression is found to be used to predict the oocyte viability and correlate with the pregnancy outcomes of IVF/ICSI^45, 46^. Our findings demonstrate that GOLPH3 expression may reduce cumulus granulosa cell apoptosis, enhance egg development ability and increase blastocyst formation. Since the cumulus granulosa cells that are removed during ICSI exhibit the strongest association with oocytes, detection of GOLPH3 expression in cumulus granulosa cells may indirectly reflect the developmental ability of ooctyes. It is considered that GOLPH3 may be serve as a biomarker for the egg quality and used to screen top-quality eggs and embryos, which facilitates the success of ICSI pregnancy. In addition, the combination of analysis of GOLPH3 expression in cumulus granulosa cells and time-lapse incubation for embryo culture is useful to further validated the potential of GOLPH3 expression in the selection of top-quality eggs^47, 48^.

In summary, the results of this study demonstrate that GOLPH3 may be involved in the apoptosis of cumulus granulosa cells, which may correlate with oocyte maturation and egg development. The quantification of GOLPH3 expression in cumulus granulosa cells may facilitate the selection of top-quality eggs and embryos, the prediction of the clinical pregnancy outcomes of ICSI, and the increase of the pregnancy rate. Further studies to investigate the mechanisms underlying the effect of GOLPH3 on the development ability of oocytes seem justified.

## Subjects and Methods

### Ethical approval

This study was approved by the Ethical Review Committee of Fujian Provincial Maternity and Children’s Health Hospital of Fujian Medical University (approval number: 2013–011). Written informed consent was obtained from all subjects involved in this study following a detailed description of the purpose of the study, and all subjects involved in this study agreed to publish related demographic and clinical features. All experimental procedures described in this study were conducted in accordance with international, national and local laws, regulations and guidelines.

### Subjects

A total of 119 women who were admitted to the Unit of Human ART, Fujian Provincial Maternity and Children’s Health Hospital of Fujian Medical University (Fuzhou, China) for ICSI during the period from April 2012 through June 2014, due to their husbands’ suffering from oligospermia, asthenospermia, teratozoospermia or oligoasthenoteratozoospermia, were enrolled in this study.

The inclusion criteria involved (1) women that were aged less than 35 years and underwent ICSI for the first time; (2) women with indications of ICSI; (3) women undergoing a gonadotropin-releasing hormone agonist (GnRHa) long protocol for COHS; (4) women with regular menses, of 25 to 35 days for each menstrual cycle; (5) women with normal baseline sex hormone levels, and without hormone therapy during the latest three months; (6) women with BMI between 16 and 29.38 kg/m^2^; (7) women with normal uterus (no hysteromyoma or endometrial polyp); and (8) informed consent was signed.

Women with the following disorders: (1) age of >35 years, and/or baseline follicle-stimulating hormone (FSH) level of >10 mIU/ml; (2) a history of surgery for ovary or chemoradiotherapy; (3) development of premature ovarian failure or hypo-ovarianism; (4) administration of steroid hormones during the recent three months;(5) development of endometrial lesions or endocrine diseases; (6) presence of polycystic ovary syndrome (PCOS), ovulatory disorder, hyperprolactinemia, thyroid dysfunction, endometriosis, sterility of unknown origin, immune sterility or a history of pelvic tuberculosis; (7) those with ovulation-stimulation treatment, or canceling fresh transfer cycles during the recent three months; (8) those with hydrosalpinx; (9) those with contraindications of ovulation-stimulation drugs; (10) those suffering from epilepsy, HIV infection, diabetes or liver/kidney/lung diseases; (11) contraindication for pregnancy; (12) a history of drug or alcohol abuse; (13) vaginal bleeding of unknown causes; (14) presence of tumors in ovary, breast, pituitary or hypothalamus; (15) genital malformation; (16) chromosomal abnormality or (17) drug allergy; or their husbands with the following disorders: (1) severe oligospermia, (2) obstructive azoospermia, (3) retrograde ejaculation, (4) anejaculation, or (5) chromosomal abnormality, were excluded from the study.

The demographic and clinical characteristics of all participants were recorded, including age, BMI, duration of sterility, cause of sterility, baseline sex hormone levels, baseline antral follicle count, mean gonadotrophin (Gn) level and mean endometrial thickness. One to four cumulus granulosa cells in the peripheral region of a single egg and all remaining cumulus granulosa cells were captured from each subject for the subsequent experiments.

### COHS protocol and egg retrieval

A long COHS protocol utilizing GnRHa, Gn and human chorionic gonadotropin hormone (hCG) was employed in this study. (1) Short-acting GnRHa was injected at a dose of 0.03 to 0.1 mg since the midluteal phase until one day prior to or at the day of hCG injection; (2) Serum FSH, luteinizing hormone (LH) and estrogen (E_2_) concentrations were determined and transvaginal B-mode ultrasonography was performed 10 to 14 days post-injection with GnRHa, and Gn (FSH/hMG) therapy was initiated after pituitary down-regulation achieved the assigned criteria; (3) Criteria of pituitary down-regulation: a follicular diameter of 5 to 10 mm and an endometrial thickness of 5 mm or less, serum LH of <5 U/ml, FSH of <5 U/ml and E_2_ of <50 pg/ml; (4) Gn was given at an initial daily dose of 100 to 375 U, and transvaginal B-mode ultrasonography, detection of serum E_2_ concentration and monitoring of follicular development were performed 4 to 5 days post-treatment with Gn. The dose of Gn was adjusted according to the follicular development until the day of hCG injection, and then follicular development was monitored and serum E_2_ concentration was detected once daily or once every other day; (5) If B-mode ultrasonography detected a dominant follicle with a diameter of 19 mm or greater, and 2 to 3 dominant follicles with a diameter of 17 mm or greater, and serum E_2_ concentration reached 200 pg/ml for each dominant follicle, the injection of FSH/hMG was terminated, and hCG was injected at a dose of 5,000 to 10,000 U at the night of the day. Eggs were sampled 34 to 36 h post-injection. In addition, the dose of hCG was reduced for those at risk of ovarian hyperstimulation syndrome (OHSS); (6) For women without ovulation, with irregular ovulation or inconvenient for ovulation monitoring, oral contraceptives were administered on days 3 to 5 of the menstrual cycle, once daily for successive 21 to 25 days, while oral contraceptives were administered at any time (after exclusion of pregnancy) or since days 3 to 5 of the menstrual bleeding after progesterone withdrawal in women with oligomenorrhea or amenorrhea. On day 16 post-administration with oral contraceptives, transvaginal B-mode ultrasonography was performed, and GnRHa therapy was initiated.

Eggs were retrieved through transvaginal puncture with single- and double-lumen needles guided by B-mode ultrasonography at a negative pressure of 100 to 120 mmHg, and all follicles were retrieved. All eggs captured were used for ICSI fertilization.

### ICSI treatment

Oocyte corona cumulus complexes (OCCCs) were obtained using the egg retrieval technique, transferred to 200 μl hyaluronidase solution, and gently aspirated in and out of a Pasteur pipette until the exfoliation of cumulus cells. Then, the eggs containing corona radiata cells were immediately transferred to Gamete medium (Vitrolife; Gothenburg, Sweden), and corona radiata cells were removed from eggs with a Pasteur pipette until the complete exposure of eggs. Subsequently, hyaluronidase was completely washed away, and eggs were cultured in a triple-gas bench top incubator (Cook Medicals; Bloomington, IN, USA) following the assessment of egg maturation based on extrusion of the first polar body. If eggs are at a germinal vesicle (GV) or germinal vesicle breakdown (GVBD) stage, these egg have not completed the first meiotic division, and are identified immature, which cannot be used for ICSI. If the first polar body is present in perivitelline space, the eggs are considered at metaphase II and identified mature, which can be used for ICSI.

ICSI was performed as described previously. Briefly, a drop of polyvinyl pyrrolidone (PVP) was added in the center of the petri dish, which was surrounded by the Gamete medium containing HEPES. The sperm was transferred to PVP, and eggs were placed in the Gamete medium. Active sperm with forward movement ability was used for ICSI. The sperm tail was pressed with an injection pipette, and a single sperm was sucked into the injection pipette from the tail and placed approaching the pipette tip. Oocytes were fixed with holding pipettes (the first polar body was placed close to the 6 or 12 o’clock position), and the injection pipette penetrated through oocytes. Then, the sperm was injected into the cytoplasm of the oocyte, and eggs were transferred to IVF-30 culture medium (Vitrolife; Gothenburg, Sweden) and incubated in a triple-gas bench top incubator to complete the fertilization process.

### Embryo culture and transfer

The zygote was rinsed with fresh G1 culture medium (Vitrolife; Gothenburg, Sweden), and then transferred to G1 medium droplets one by one. Fertilization was observed one day after embryo culture under a microscope at a magnification of ×400. Embryos containing double pronuclei and two polar bodies in the perivitelline space were identified as normally fertilized embryos, while zygotes with a single pronucleus, three or more pronuclei, or without a pronucleus were defined as abnormal fertilization or infertilization. Embryo development was observed 2 and 3 days post-culture and embryos were graded as following. Cleavage-stage embryos were graded with a 1 to 4 scoring system, based on speed of embryo development, fragmentation, cell symmetry, and number of blastomere, and Grade 1 and Grade 2 cleavage-stage embryos were identified as “top quality” embryos, while Grade 1 to 3 cleavage-stage embryos were defined as transferrable/frozen embryos. Two embryos with the highest scores were transferred to the embryo glue, and the remaining transferable embryos were transferred to G2 blastocyst culture medium (Vitrolife; Gothenburg, Sweden) for a further incubation until blastocyst formation. After embryo culture for 5 to 6 days, if blastocysts were produced, top-quality blastocysts were screened using a 1 to 6 scoring system based on the degree of expansion and hatching status of each blastocyst, with Grade 3 to 6 blastocysts graded according to the inner cell mass (ICM) and trophectoderm (TE), and cryopreserved by vitrification.

Embryo transfer was performed 72 h after egg retrieval, guided by transvaginal B-mode ultrasonography. For women aged less than 35 years, 2 embryos were transferred at the first cycle and 3 embryos were transferred at the second and higher cycle, while 3 embryos were transferred for those at ages of over 35 years, as recommended by the Ministry of Health, People’s Republic of China. Following embryo transfer, the women were stayed in bed for 0.5 h. At the day of egg retrieval, progesterone was administered by intramuscular injection at a daily dose of 60 to 100 mg for luteal phase support.

Urine hCG assay was performed 14 days or serum hCG levels were measured 14 to 16 days after embryo transfer to identify pregnancy. Luteal phase support was continued to 30 days in women positive for pregnancy testing, and the detection of gestational sac and fetal heartbeat by B-mode ultrasonography was considered successful clinical pregnancy. All pregnant women were followed up, and 6 to 7 weeks of gestation was defined as clinical pregnancy, and 10 to 12 weeks of gestation as ongoing pregnancy.

### Collection of cumulus granulosa cells and sperm

**Figure 7:**
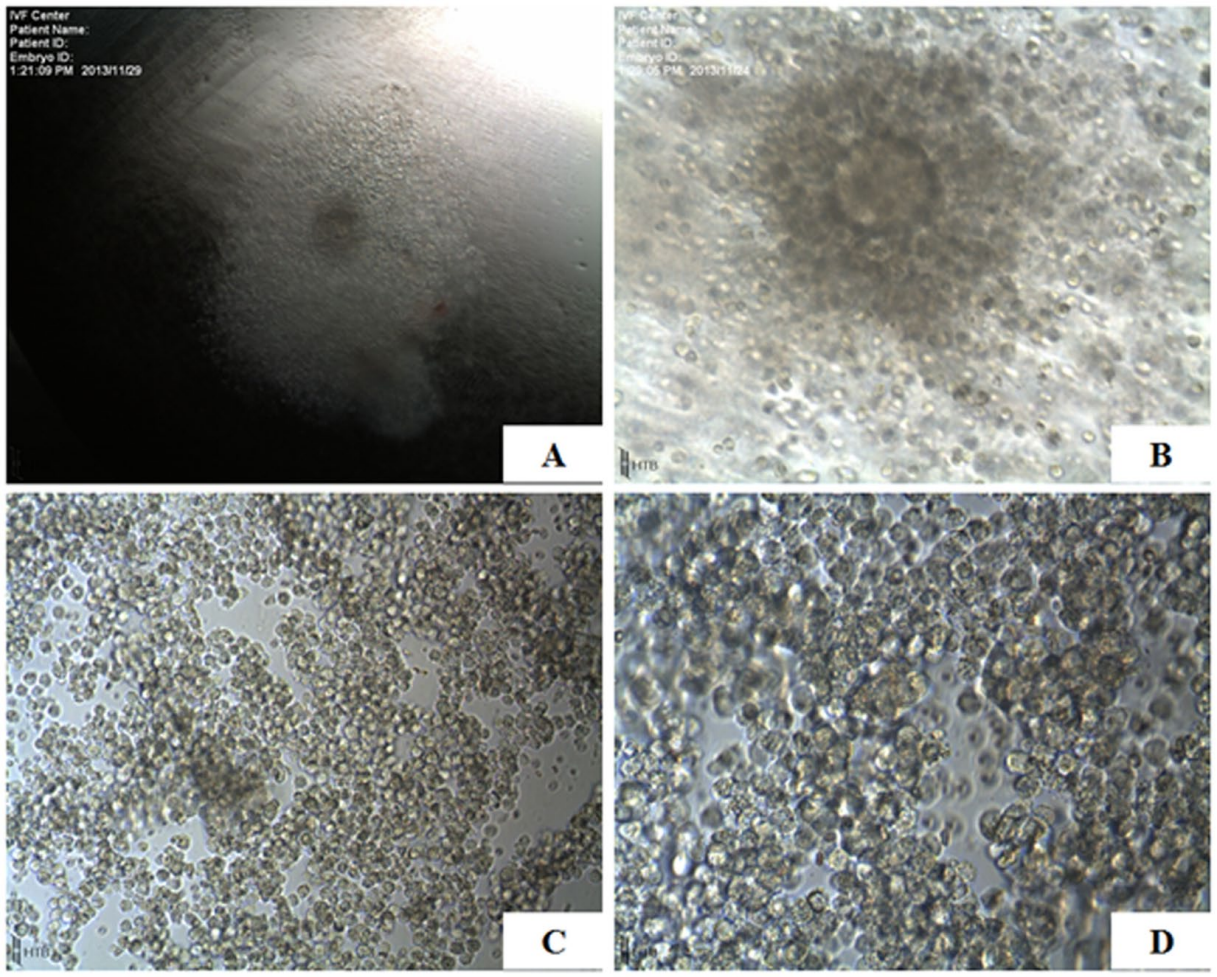
Oocyte corona cumulus complex, (OCCCs) (**A** and **B**) and cumulus granulosa cells (**C** and **D**). A, ×4; B, ×10; C, ×10; D, ×40.

During egg retrieval, each follicle was punctured, and OCCCs (Fig. 7) were selected under an anatomic microscope. According to the number of total follicles, 1 to 4 OCCCs were collected from each subject. During ICSI procedures, cumulus granulosa cell clusters were digested in 0.1% hyaluronidase, and pipetted into a single cell. All cumulus granulosa cells in the peripheral region of each egg were transferred to 0.5 ml centrifuge tubes, and centrifuged at 200 g for 5 min. The supernatant was discarded, and the sediment was stored at −80 °C for the subsequent measurements.

Then, the cumulus granulosa cells surrounding the remaining eggs were collected with a Pasteur pipette, transferred to two divided 0.5 ml centrifuge tubes, mixed evenly in 200 μl PBS, centrifuged at 200 g for 5 min, and the supernatant was discarded. After washing twice in PBS, a tube of cumulus granulosa cells was store at -80 °C, and another tube of cells was re-suspended in PBS and adjusted to a 1 × 10^6^ cells/ml density for the subsequent experiments.

Following fertilization, all remaining sperm samples were mixed evenly with cryoprotectant at a 1: 1 ratio, and cryopreserved at −196 °C for detection of apoptosis.

### Immunohistochemistry

Cumulus granulosa cells were fixed in 4% paraformaldehyde at room temperature for 20 min, microwave-antigen retrieved, blocked in 3% H_2_O_2_ at room temperature for 10 min, rinsed with PBS and incubated in rabbit anti-human GOLPH3 antibody (1:100 dilution) at 4 °C overnight. Then, cells were rinsed with PBS, incubated in HRP-conjugated anti-rabbit secondary antibody at room temperature for 30 min, rinsed with PBS, and visualized with DAB. Cell nuclei were counterstained with hematoxylin, differentiated 0.1% hydrochloric alcohol, dried and enveloped with neutral resin, while cells incubated in PBS served as negative controls. Pale yellow cells were defined as mildly positive (+), brown cells as positive (++), and chocolate-brown cells as strongly positive (+++). Five fields of vision were randomly selected from each sample, of 100 cells counted in each field, and the proportion of positive cells was calculated. The immunohistochemical staining of GOLPH3 in cumulus granulosa cells was graded using the scoring system described previously^49, 50^.

### Detection of apoptosis

The apoptosis of cumulus granulosa cells was determined using HE staining. Briefly, cumulus granulosa cells were re-fixed in 4% paraformaldehyde at room temperature for 1 h, washed three times in PBS, stained with hematoxylin for 5 min and rinsed with flowing tap water for 5 min. Following de-colorization once or twice in acid alcohol solution, cells were rinsed with flowing tap water for 4 min, placed in ammonium hydroxide water solution (Scott; New Orleans, LA, USA) for 1 min, rinsed with flowing tap water for 4 min, stained with eosin Y alcohol solution for 2 min, rinsed with flowing tap water, placed in 70% methanol for 3 min, dried and enveloped with neutral resin. Apoptotic cells were observed under an oil microscope. Four to six fields of vision were randomly selected from each sample, and 200 cells were observed to estimate the apoptotic rate. The nucleus of healthy cells had a normal morphology, and weak and even staining, while apoptotic cell nuclei had deep staining, an irregular morphology, and even strongly stained apoptotic body.

The apoptosis of sperm and granulosa cells was detected with a PE Active Caspase-3 Apoptosis kit. Briefly, 100 μl of cell suspensions was mixed evenly in cool PBS, centrifuged at 1,200 r/min for 5 min, washed twice in PBS, adjusted to a cell density of 1 × 10^6^ cells/0.5 ml with the Cytofix/Cytoperm Solution, and placed on ice-bath for 20 min. Cells were then centrifuged, and the supernatant was discarded. The sediment was washed twice in perm/wash buffer at room temperature. Then, each sperm sample was suspended in 100 μl of perm/wash buffer, incubated with 20 μl of anti-Caspase-3 antibody for 30 min, while those added with the same volume of perm/wash buffer served as blank controls. Cells were then washed with 1 ml of perm/wash buffer, and formulated to cell suspensions with 0.5 ml of perm/wash buffer. Apoptosis was detected on a FACS Calibur flow cytometer.

### qRT-PCR assay

*GOLPH3 mRNA* expression was quantified using qRT-PCR assay. Total RNA was extracted from granulosa cells with the High Pure RNA Isolation Kit, and transcribed reversely into cDNA using the HiFi-MMLV First-Strand cDNA Synthesis Kit. qRT-PCR assay was performed in a 20 μl reaction system containing 10 μl of 2 × UltraSYBR Mixture, 0.4 μl forward (5′-CTCGGACGTCCTGGAGAATG-3′) and reverse (5′- GCCCACAGAACCTCATTGGT -3′) primer each (10 μM each), 2 μl cDNA template, and 7.2 μl ddH_2_O, under the following conditions: at 95 °C for 10 min, followed by 45 cycles of at 95 °C for 15 s and at 60 °C for 60 s, while *GAPDH* (F: 5′-GGAAGGAAATGAATGGGCAGC-3′, R: 5′-TAGGAAAAGCATCACCCGGAG-3′) served as an internal control. The relative quantity of *GOLPH3 mRNA* expression was calculated using the 2^−△△CT^ method.

### Western blotting analysis

GOLPH3 protein expression was determined with the Western blotting assay. Total protein was extracted from granulosa cells, and quantified with the BCA assay, and separated with SDS-PAGE. Then, gels were electro-transferred to PVDF membranes, which were placed in the blocking solution at room temperature for 1 h. The membrane was incubated in rabbit anti-human GOLPH3 polyclonal antibody (1:300 dilution) at 4 °C overnight, washed three times in 1 × TBST at room temperature, of 10 min each time, incubated in HRP-conjugated goat anti-rabbit secondary antibody (1:3,000 dilution) in darkness at room temperature for 60 min, and washed three times in 1 × TBST at room temperature, while β-actin served as a loading control. PVDF membranes were dried, and visualized with the ECL reagent. The GOLPH3 protein expression was quantified using the software Image J.

### Statistical analysis

All measurement data were described as mean ± standard deviation (SD), and all count data were expressed as proportions. Comparisons of measurement data were performed with independent sample *t* (homogeneity of variance) or *t*′ test (heterogeneity of variance), and Mann-Whitney *U* test was employed for non-normally distributed data, while differences of proportions were tested for statistical significance with chi-square test or Fisher’ exact test. The associations of pregnancy with GOLPH3 expression, apoptosis of cumulus granulosa cells and ICSI clinical outcomes were examined with Pearson correlation analysis. All statistical analyses were performed using the statistical software SPSS version 13.0 (SPSS, Inc.; Chicago, IL, USA), and a *P* value < 0.05 was considered statistically significant.

## Electronic supplementary material

Supplementary Information
