# Supplementary material for: Effect of GOLPH3 on cumulus granulosa cell apoptosis and ICSI pregnancy outcomes
Source: Sci Rep. 2017 Aug 11;7:7863. doi: 10.1038/s41598-017-08343-w (PMC5554214; doi:10.1038/s41598-017-08343-w)

Effect of GOLPH3 on cumulus granulosa cell apoptosis and ICSI pregnancy outcomes

Dianliang Lin[1¶*], Jing Ran[3], Suqin Zhu[1,2¶], Song Quan[2*], Baofeng Ye[1], Aili Yu[1], Yuefan Kang[1], Yuan Lin[1]

1 Fujian Provincial Reproductive Medicine Center, , No. 18 Daoshan Road, Fuzhou City, Fujian Province, 350001, China.

2 Center for Reproductive Medicine, Department of Fujian Provincial Maternity & Children Hospital, Affiliated Hospital of Fujian Medical University Obstetrics and Gynecology, Nanfang Hospital, Southern Medical University, 1838 North Guangzhou Road, Guangzhou City, Guangdong Province, 510515, China.

3 Department of Gynecology and Obstetrics, the First Affiliated Hospital of Xiamen University, 55 Zhenhai Road, Xiamen City, Fujian Province, 361003, China.

¶ These authors contributed equally to this work.

* Correspondence. Song Quan, Center for Reproductive Medicine, Department of Obstetrics and Gynecology, Nanfang Hospital, Southern Medical University, 1838 North Guangzhou Road Guangzhou City, Guangdong Province, 510515, China. E-mail: quansong@smu.edu.cn or quansong2008@gmail.com; or Dianliang Lin, Fujian Provincial Reproductive Medicine Center, Fujian Provincial Maternity & Children Hospital, Affiliated Hospital of Fujian Medical University, No. 18 Daoshan Road, Fuzhou City, Fujian Province, 350001, China. E-mail: mqldl@163.com.

Figure. S1 qRT-PCR and Western blotting assays detect GOLPH3 expression in cumulus granulosa cells. A, Agarose gel electrophoresis of total GOLPH3 RNA. 1, Non-pregnant group; 2, Pregnant group; B, Western blotting analysis detects GOLPH3 protein expression in cumulus granulosa cells, and β-actin serves as a loading control. 1, Non-pregnant group; 2, Pregnant group.

A

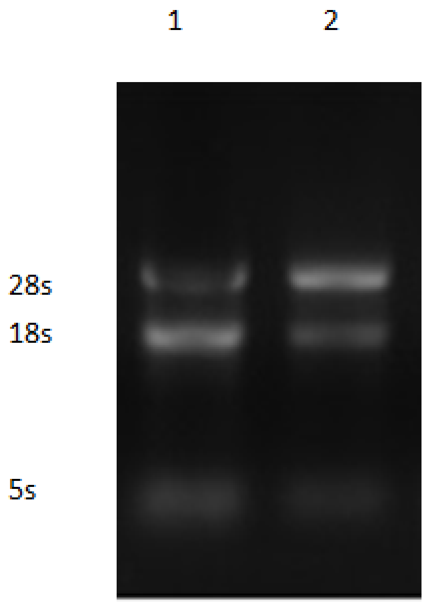

B

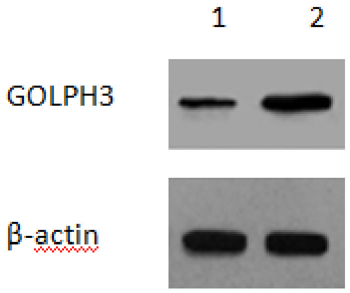

Supplement: Supplementary file 1 — Supplementary Information [file 41598_2017_8343_MOESM1_ESM.pdf]
